# Dr. Alder's Proofs of the Proximate Causes of Diseases from the Practice of Physic

**Published:** 1809-12

**Authors:** Thomas Alder


### Dr. Alder's *Proofs of the proximate Causes of Diseases from the Practice of Physic.*

( In Continuation. )

It will be easy for Gentlemen to raise a formidable opposition against me here, for three reasons, even supposing my theory is right: for, in the first place, I can scarcely prefer any practice whatever, (however right it may be) without having somebody against it; and in truth I fear it must be owned, that if I adopt the most efficacious and perfect practice possible to be used for all cases, and for every case, I shall do infinitely more than anybody else has done; while at the same time this (as I said before) is that part of my work for which I feel myself adequate. In the second place, supposing (which is supposing a great deal,) that I select the proper practice in every instance, I may yet err *in connecting this practice with my theory.* But, in the third place, I apprehend it is very possible for me to select wrong practices, and yet connect my theory with them; just in the same way as most who have gone before me have acted, when they have hung many right practices upon a wrong theory. And I the rather make these observations, because my opponents, who have hitherto generally confined themselves to secret murmuring and *insidious practices,* are most likely now to come forth with the old trite but true maxim of "judge of a tree by its fruits," and, by *misapplying it,* bring reproach upon a theory, when error, if any, *lies only in its application.*

*plication.* It is clear that my theory, if good, will not lead to bad practices; but, at the same time, it is equally obvious, that a person by blundering *may attach bad practices to a good theory.*

After these observations, I shall beg to proceed; and shall use all the caution and diligence I can.

*Bleeding.*—People seldom think of bleeding in fever in this country, except to check mere arterial action, *i. e.* increased action of the arteries without any inflammatory tension, or apoplectic state; and this is a purpose for which it is least wanted; and its employment here must, of course, appear somewhat of a doubtful nature. But as in other countries, (and these warm ones too as well as cold ones,) and occasionally in this, there often is great occasion to use it for other purposes, so in them it often appears more successful. Dr. Chisholme, for instance, says, that (from many trials) he found it the only efficacious remedy to remove a dangerous congestion in the lungs, *though he used it in the cold stage when the pulse was very feeble, and the arterial action very languid.* And I think it fortunate, that, for further information respecting this unusual and apparently critical use of it, I have it in my power to refer to a living and British author; but, at the same time, I cannot but think that this author has here, by his practice as well as theory, done much to destroy what he said in other places against bleeding; for, if Dr. Chisholme has often safely and efficaciously used it in the cold stage, (and from his own account I have no doubt he has,) I should, in *many* cases of yellow fever, as well as others, think it equal to *mercury*, and should regard only as a *memento* what he says of its ultimate bad effect where the blood drawn was red, and threw up an inflammatory crust.

Cleghorn used bleeding in remitting and intermitting fevers in a warm climate, very generally and successfully; but he had but little hope from it, *except on the first attack*; and he employed it in the beginning of the hot fit. Dr. Hillary and Dr. M'Lean, respectively, from their practice in remitting fever in the West Indies, speak of bleeding in the same way; and Dr. Rush, and many others in America, bear the same testimony. Sydenham speaks in the same way of it in England, Sir John Pringle in the Netherlands, and Prosper Alpinus in Egypt. Most of these are explicit in their declarations, that it must be used before the *third* day, and often sooner, just as is the case in pneumonia. They most of them used it to abate and pre-

 vent vent high and real inflammatory symptoms and congestions. But Sydenham once used it with instant success, when all the powers of vitality were oppressed, and nearly extinct, (evidently, in my judgment, from a congestion in the lungs,) the skin being cool. And Dr. Rush used it with success, whenever the pulse was *tense,* whether it was preternaturally slow or strong, and whether it was in the exacerbation or remission; though he most usually bled in the exacerbation. Dr. Rush seemed to consider *tension* of the arteries as the principal circumstance indicative of the propriety of bleeding. Dr. Rush often drew more than one hundred ounces of blood in seven days.

The greater part of those who practised in France too, in that dreadful plague which scourged Marseilles, found bleeding very efficacious.

Yet, after all these examples of the efficacy of bleeding in the remitting fever, whether called contagious or not, we find an author, (Dr. Thomas,) in treating of it, say, "*In every instance almost* in which bleeding has been adopted, it has proved *highly pernicious,* by inducing a state of extreme debility, under which the powers of life soon became extinct."

It is true, Dr. Lind, Dr. Clarke, and Dr. Chisholme, several practitioners in the West Indies, and in the United States of America, and some of those who practised at Marseilles, have been against bleeding in remitting fevers; but when other gentlemen have found bleeding very useful in the very same fevers, we must conclude that it is useful, and seek some reasons to account for their saying it is not so.

1st. It might not appear of much, or any efficacy, upon the whole, in the cases in which they tried it. But people who were afraid of a remedy would not like to try it, except in desperate cases, and often when others had failed, or it was too late for it to be beneficial.

2dly. Almost all these people had a prejudice against bleeding; they thought it *must* increase debility. And some of them, at the same time, had favourite remedies to recommend.

But the instances in which bleeding has *appeared* to do harm really are very few: and indeed they could scarcely be otherwise than so; for when a man *thought* bleeding had done harm, he would seldom venture to try it again. And if we subtract from these, those in which it was only *thought* to do harm; and again from these, those in which it was employed *contrary to the rules laid down for its exhibition,*

*hibition*, we shall most probably have but a very small remainder.

It must be acknowledged, that *venæsection* is a practice which, at first glance, palpably seems to result from my theory, (though the limitations of it, according to the same theory, are more numerous than one might imagine.) And I think it must at the same time be admitted, by those who are extensively conversant with these, that both *dissections* and *practice* amply confirm its general utility, while the symptoms *accurately considered* do not in reality afford any thing against it. It is a difficult point, however, to specify all the limitations of it.

*The Tournequet.*—Mr. Kellie says he has found the application of the tournequet prevent or put a stop to the cold fit of an intermittent in many instances. And a gentleman, in Dr. Coxe's Philadelphia Register, informs us, he has found it serviceable in typhus. But Mr. Kellie used it to compress the artery, and the other gentleman to check the blood in the vein. It appeared to me (before I saw what this gentleman had said) that Mr. Kellie's success was owing, in a great measure, to his compressing *the vein*; and I find my sentiments are strengthened by those of one of our best anatomists: for I asked him, "Do you think it *possible* to compress the femoral artery without, *in some degree*, compressing the vein?" And his answer was, "*In some degree?*—No, certainly not." I then said, "As the tournequet is commonly used, do you think the blood is stopped in the vein as much as in the artery, the vein being more *compressible?*" and to this he replied, "When the tournequet is used, I think blood usually must be more checked in the vein than in the artery, the vein being more compressible; but there sometimes is a *lusus naturæ* in the veins."

But the tournequet thus used, must remedy, in the vital parts, both arterial depletion and venous plethora, as well as pulmonic congestion; (that is, all the causes of the bad symptoms.)

Several unpleasant effects, however, have followed the use of the tournequet in some cases. But it is more difficult to account for the bad effects of this agent than the good ones. If the vein be not sufficiently compressed, or if the artery be compressed too much, or too long, or the artery be diseased, &c. &c. bad effects must arise in a very evident way.

*Mercury.*—Mercury seems almost a universal remedy; and it has been found useful in most of the diseases which

I attribute to impeded pulmonary transmission, as well as particularly in fevers. Nay, it seems most efficacious to prevent them: its powers in preventing fevers have been established some years; and Mons. Portal it seems has found it very successful in preventing most of the other diseases spoken of. And it is worthy of notice, that Mons. Portal has proved there is as strict an affinity in the *predisposition to those diseases which arise principally from an impeded pulmonary transmission,* as we know there is between these diseases themselves. He exhibited mercury, however, under the idea that some syphilitic taint was the ground of them; which idea I think, in very many instances, is not correct.

Mercury seems to relieve internal congestions, (in several stages, and from some different causes,) and to prevent them. It relieves many congestions in the liver, spleen, and mesentery, and also in the head. It is evident too from the change in symptoms which has often followed its use, that it relieves *some* congestions in the lungs. The question is, *how it does all this?* It evidently drives the fluids from the internal to the external parts, and opens all the excretories, and lets them off, *keeping the stomach and bowels in good order.* For this purpose, different articles are required according to circumstances. They cannot be got in good order by tonics, without they are cleared of all foul contents. Agreeably to that aphorism of Hippocrates, "the more you nourish a foul body the more you injure it." And they cannot be kept in good order without the body is so managed as not to load them with foul contents. Dr. Hamilton, and several others, have shewn the great importance of attending to the state of the intestinal canal in the diseases in question.—I shall first inquire how a disordered state of the intestinal canal can do harm; and then point out the remedies calculated to obviate or prevent it. When the intestinal canal is disordered, much gas will usually be generated, which will partly distend it, and partly be obtruded into the lacteals, (from the peristaltic contractions of the intestines,) and thus conveyed into the sanguiferous system, increasing congestions, first in the lungs, and afterwards in the whole body. In this I am supported, both by dissections and symptoms. An apoplectic state may arise from it very suddenly. But when air only distends the intestines, it must push up the diaphragm, and greatly impede respiration. From the great quantity of nervous matter, however, distributed upon the intestinal canal, a disordered state of it (I am willing to add) must depress the the nervous system, and by that means act essentially upon the body: but, upon the whole, I do not think that this action of it is so dangerous as the two preceding.

To remove this disordered state of the intestinal canal, *emetics, purgatives, tonics, wine and bark, pure air, pleasing the mind, the cold bath, and astringents, &c.* have been used according to circumstances. But many of them are often used for another purpose, and well demand a *particular* consideration. And emetics do not seem well adapted to bad fevers in a hot climate, as they render the stomach so irritable.

*Emetics.*—Emetics seem very serviceable in some febrile diseases, in cold and temperate climates. They have done much good, given some hours previous to the cold fit of intermittents; and in nauseating doses they are very efficacious, when there is a phlegmonic inflammation, or a strong tendency to it, in the system, with fever, as often happens to lying-in women, and as exists after catching cold, &c. But for an action purely febrile, other remedies are much better; and they often hurt the stomach, even in this country. Dr. Cullen, (it seems, following his theory,) made them by far too fashionable. When they do good, (independently of clearing the stomach,) it is by driving the fluids from the internal to the external parts, and relaxing the skin so as to let them off. Also in inflammatory cases *(not purely febrile)* they do good by relaxing the inflammatory contraction of the arteries.

*Pleasing the mind.*—The utility of this is well known, and its prodigious power will be appreciated by reading Falconer's and Tissot's Dissertations before spoken of. The first mentions that sailors, who had been confined for months with the scurvy, recovered most rapidly when they expected an engagement; and, when the battle came on, they were all up and at their fighting quarters; and that at the siege of Breda, the garrison all became as bad almost as they could be with this disease, but that, on receiving assurance of speedy relief, and some coloured water, or some such thing, alleged to be a sovereign remedy, they all recovered cheerfulness and health most perfectly, tho' but a little before *their muscles were hardened and contracted,* and they were not able to move. And the scurvy is a disease of all others the most adapted to prove the great effect cheerfulness has upon the *sanguiferous* system, because almost the whole of the disease lies in this system, and comparatively but very little of it in the nervous. The almost rotten state of the body too, and the rigidity and contraction of the muscles which constitute it, cannot be removed so soon and perfectly as they were here, but by remedies which have *a prodigious effect upon the sanguiferous system*; and if these remedies act in the first place *upon the nervous*, we can in these cases (as well as several others) only infer, that the nerves are *the media* through which they operate.

The question then is, how does a pleasant state of mind act upon the blood-vessels? It no doubt instantly affects almost every part on which there is distributed nervous matter, and in this way produces much effect. But still it acts greatly through the medium of respiration. For, as far as my observation goes, the respiration becomes free and perfect as the mind becomes cheerful, and impeded as it becomes depressed.

As, however, to the mind, in fevers, (from the converse of this holding good, that is, from the mind being distressed and oppressed, as respiration is so) it often is not easy to please the mind without previously making the respiration good; which is saying, that mere mental remedies often will not act. In some cases too we have no cheering prospects to offer sufficient to make much impression. In both these cases, however, we may often induce a grateful state of the mind by operating on the body; in the former, by remedies particularly calculated to remove the disease from the respiratory as well as other organs; and, in the latter, sometimes by WINE, OPIUM, &c. And Dr. Falconer, in the same books, judges as I do, that a great part of the beneficial effects of these remedies, in certain cases, is owing to their inducing a grateful state of mind. It is almost useless now, perhaps, to add, that through the mind they affect respiration. But it certainly is not in all cases, that either opium or wine will please the mind; I believe they very seldom will, where, from some particularly disordered state of the body, they do harm to it. But, I think, where they do no harm to the body, they always will—where they do good, they must. This part of the disquisition may, perhaps, appear to some but little better than a string of truisms; but we shall hereafter make, I trust, many important inferences from it.

I am, &c.

THOMAS ALDER.

*Nov.* 1, 1809.

( To be continued. )

